# Granuloma annulare and possible relation to purified protein derivative administration: a case report

**DOI:** 10.1186/s13256-024-04598-w

**Published:** 2024-06-20

**Authors:** Ernest C. Lee, Cheryl A. Steffen, Minnerva E. Carroz, Christine L. Lee, Lysette A. Lee

**Affiliations:** 1 Phoenix VA Health Care System, Phoenix, USA; 2 The University of Arizona College of Medicine, Phoenix, USA; 3 Arizona State University, Tempe, AZ USA

**Keywords:** Granuloma annulare, Erythematous plaque, Annular plaque, Case report

## Abstract

**Background:**

Granuloma annulare is a noninfectious inflammatory granulomatous skin disease characterized by an erythematous or skin colored annulare plaque. The diagnosis of granuloma annulare may be challenging owing to its diverse morphology. In such cases, a correlation between the clinical findings and histologic findings are necessary.

**Case presentation:**

We report a case of granuloma annulare after purified protein derivative administration. A 56-year-old Caucasian female patient complained of mildly pruritic rashes which started on both arms and lower extremities, and eventually spread to both thighs, the left popliteal region, left upper back, and the right abdominal area. About 6 weeks prior to the eruption of the rashes, the patient had been given a purified protein derivative tuberculin skin test. Biopsy specimens revealed dermal histiocytes palisading around areas of mucin and degenerated collagen, confirming granuloma annulare. After treatment with 0.1% topical triamcinolone acetanide and 500 mg oral metronidazole, the patient’s lesions resolved.

**Discussion:**

Relatively little is known about granuloma annulare’s exact etiology. Granuloma annulare has four variations presenting as either localized, generalized, subcutaneous, or perforating and patch granuloma annulare. The clinical prognosis for granuloma annulare varies according to clinical subtypes. Proposed causal mechanisms of subcutaneous granuloma annulare include physical trauma, infections, immunizations, insect bites, diabetes mellitus, and alterations in the cell-mediated immune responses. The disease likely has an inflammatory component. Clinically, granuloma annulare may be confused with many other skin diseases.

**Conclusion:**

This case of subcutaneous granuloma annulare was reported since it is a rare dermatologic pathological condition that can be confused with other skin rash disorders. Although it is a benign self-limited disease, definitive diagnosis is important to rule out other pathologies with similar clinical appearances, such as cancer or human immunodeficiency virus (HIV) infection. Diagnostic confirmation is best made through skin biopsy.

## Background

Granuloma annulare (GA) is a noninfectious inflammatory granulomatous skin disease characterized by an erythematous or skin colored annulare plaque. “Annulare” refers to the morphology of the lesions, which, in localized forms of GA, appear as isolated, skin-colored to erythematous circinate papules and plaques. GA can also present in a generalized fashion, with disseminated GA being defined as at least ten widespread annular plaques [1]. GA is often a self-limited disorder that can affect both children and adults in all age groups, with more women affected than men. The typical lesion of GA starts as plaques and papules that have annular rings. The lesions can be seen starting at the dorsal surfaces of the hands, arms, and feet [1–3].

GA has four variations presenting as either localized, generalized, subcutaneous, or perforating and patch GA. The localized form of GA is the most common form that presents as an asymptomatic, erythematous, or skin-colored annular lesion with a border that is described as rope like with a central clearing. The generalized form, which occurs in the upper and lower extremities and in the trunk, accounts for 15% of the cases and presents as papules and plaques. The less common forms of GA include patch, perforating, and subcutaneous variants. The patch form of GA lesions can cover a large area of the skin and presents as a flat lesion. The rashes can be localized or generalized, and commonly involve the proximal extremities. In perforating GA, the lesion may sometimes resemble a pustule. It can be erythematous or a skin-colored papule that changes to an umbilicated papule with a clear white fluid discharge. A small number of patients may have pain or pruritus. Lesions may heal with residual scarring. This type of variant can sometimes be localized or with a widespread distribution. Subcutaneous GA lesions are commonly found on the anterior lower legs, hands, head, and buttocks of children. They present as a single nodule that can be deep or subcutaneous. The hallmark of a subcutaneous GA is a painless nodule on the scalp or extremities with the overlying skin appearing normal [1–3].

The history on the discovery of GA started with Tomas Colcott Fox, who first drew attention to the persistent ringed erythema [4]. Later Dr. Radcliff Crocker called it granuloma annular [5]. The exact cause on why a patient gets GA is not exactly known. However, GA has been associated with diabetes, thyroid disease hyperlipidemia, infections, and mild trauma [3, 5]. Other reported potential triggers could be related to drug eruptions, an infection, such as the severe acute respiratory syndrome coronavirus 2 (SARS-CoV-2) and other viral infections, subcutaneous injections for desensitization, vaccinations against hepatitis B, Bacillus Calmette-Guerin (BCG), human papilloma virus, diphtheria, and tetanus [6–8].

The diagnosis of GA may be challenging owing to its diverse morphology. The approach to diagnosing GA depends on the clinical presentation. The diagnosis of GA can sometimes be made on the basis of the classic clinical features of GA. In cases that are harder to diagnose, a correlation between the clinical findings and histologic findings are necessary [8].

An acquaintance with the varied histomorphology of GA is of utmost importance to render a correct diagnosis and understand the pathogenesis. Pointers toward the correct diagnosis include presence of degenerated collagen and dermal mucin. Other histologic features, such as vasculitis and eosinophil and plasma cell infiltration, may enhance the understanding of the pathogenesis of this disease. Histologically, GA presents as a focus of necrobiosis surrounded by palisading histiocytes. Mucin deposition and multinucleated giant cells are a common finding. Eosinophils, lymphocytes, and neutrophils typically invade the dermis [9]. Different subtypes of GA have various characteristic features, although the triad of degraded collagen, histiocytic infiltrate, and presence of mucin seem to be common findings across all subtypes of GA [1].

## Case presentation

**Fig. 1:**
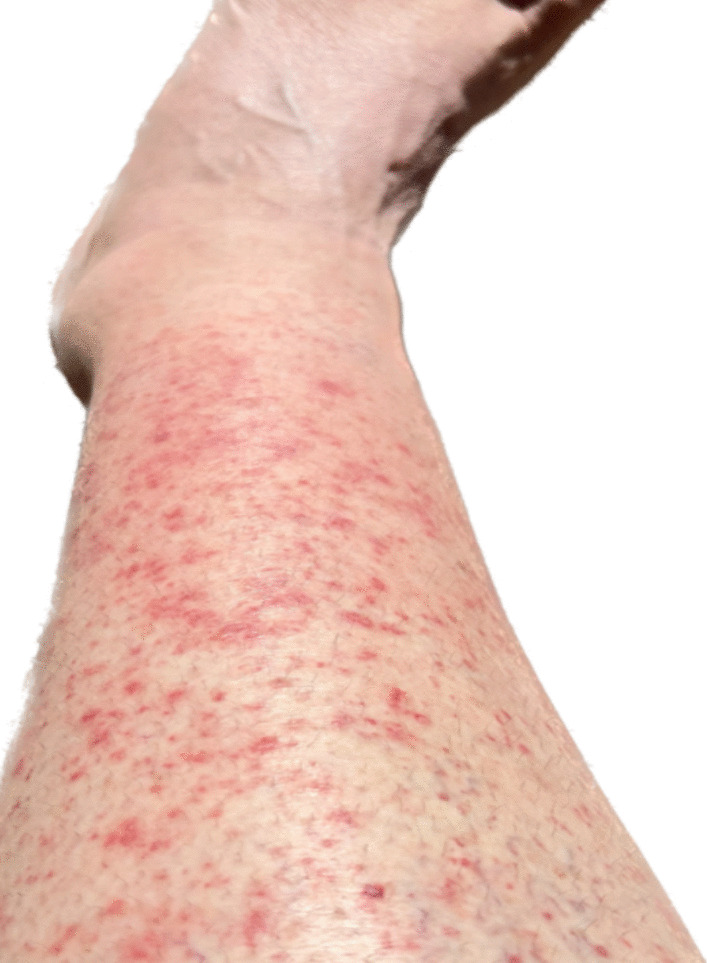
Erythematous papules on left anterior lower leg area

**Fig. 2:**
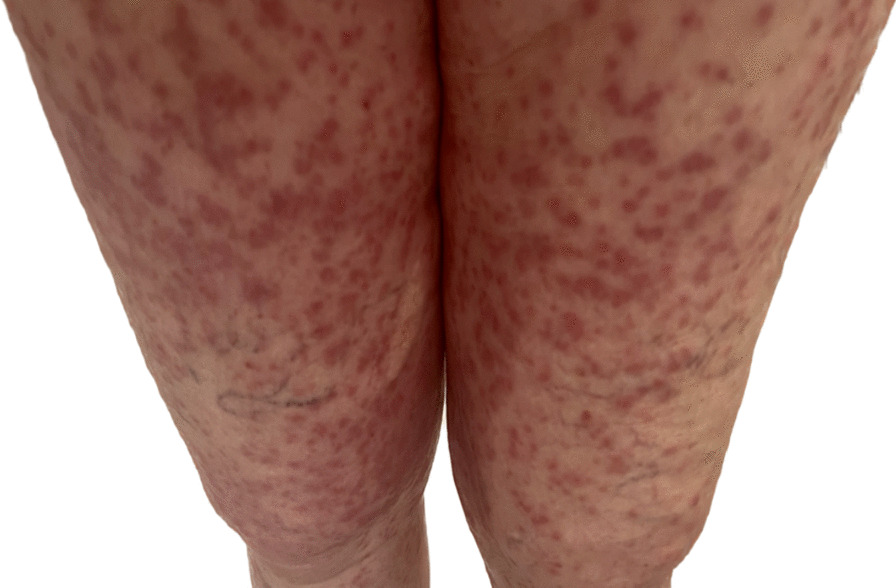
Erythematous plaques diffusely scattered on anterior thighs

**Fig. 3:**
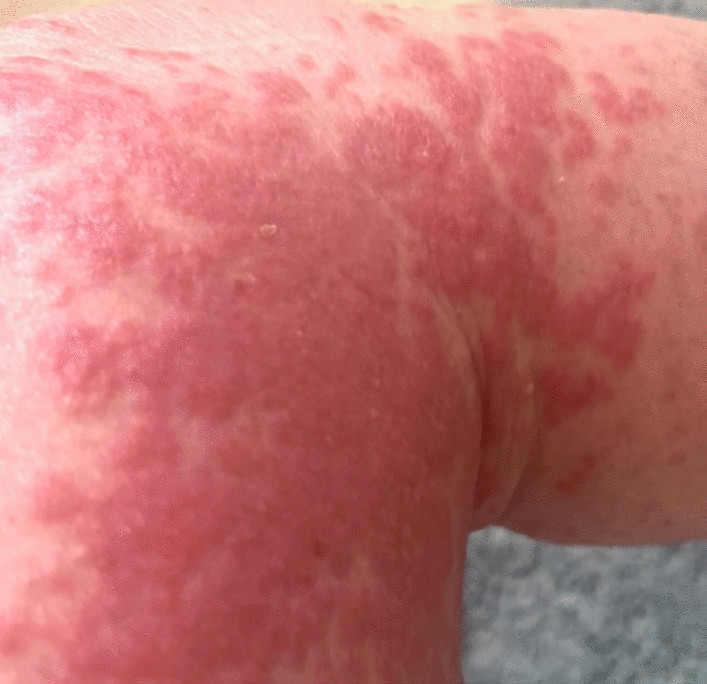
Erythematous confluent plaques on distal medial thigh and proximal medial calf

A 56-year-old Caucasian female patient was seen in an outpatient clinic with complaints of a nonspecific rashes, which had started about 1 week prior to her visit. The rashes initially started on both arms and lower extremities and were mildly pruritic. The patient self-treated with calamine lotion and diphenhydramine, which relieved the itching but did not prevent the spread of the rash. By the time she came to the office, the rashes had spread to both thighs, the left popliteal region, left upper back, and the right abdominal area. Photos of the rash can be seen in Figs. 1, 2, and 3. The rash did not involve the head. She denied any other symptoms.

About 6 weeks prior to the eruption of the rashes, the patient had been given a purified protein derivative (PPD) tuberculin skin test as part of a pre-employment medical evaluation. She did not have any symptoms of active tuberculosis, such as fevers, productive cough, unexplained weight loss, or fatigue. The patient also recalled eating orange chicken, chow mein, and crab puffs at an Asian fast-food restaurant for three consecutive days. She reported having had eaten at the same fast-food restaurant in the past without any issues. At around this time, the patient had also been in close contact with her dog.

The patient has a past medical history of heartburn treated with 20 mg of famotidine twice a day as needed, and seasonal allergies treated with 180 mg of fexofenadine once a day as needed. She denied a history of hypertrophic scarring or keloid formation or allergies to medications. She had an appendectomy in the remote past and reported a family history of basal cell carcinoma. The patient works as a registered nurse at a large medical center. She is in a monogamous relationship, drinks one alcoholic beverage once a week, and consumes caffeine daily. She denied tobacco use.

Physical examination showed multiple erythematous papules and plaques on the inferior extremities, thighs, arms, and abdominal area. The lesions were nonscaling and did not show any central clearing or umbilication.

Stool culture was negative for *Salmonella*, *Shigella*, *Campylobacter*, or *Escherichia coli*. Shiga toxin and *Clostridium difficile* toxin tests were negative. The stool was also negative for ova and parasites. White blood cell (WBC) count was normal at 7.5 × 10E3/uL. Absolute eosinophil count was slightly elevated at 0.65 K/uL (0.01–0.57). Hemoglobin, hematocrit, aspartate aminotransferase (AST), alanine aminotransferase (ALT), alkaline phosphatase, and total bilirubin were all within normal limits.

**Fig. 4:**
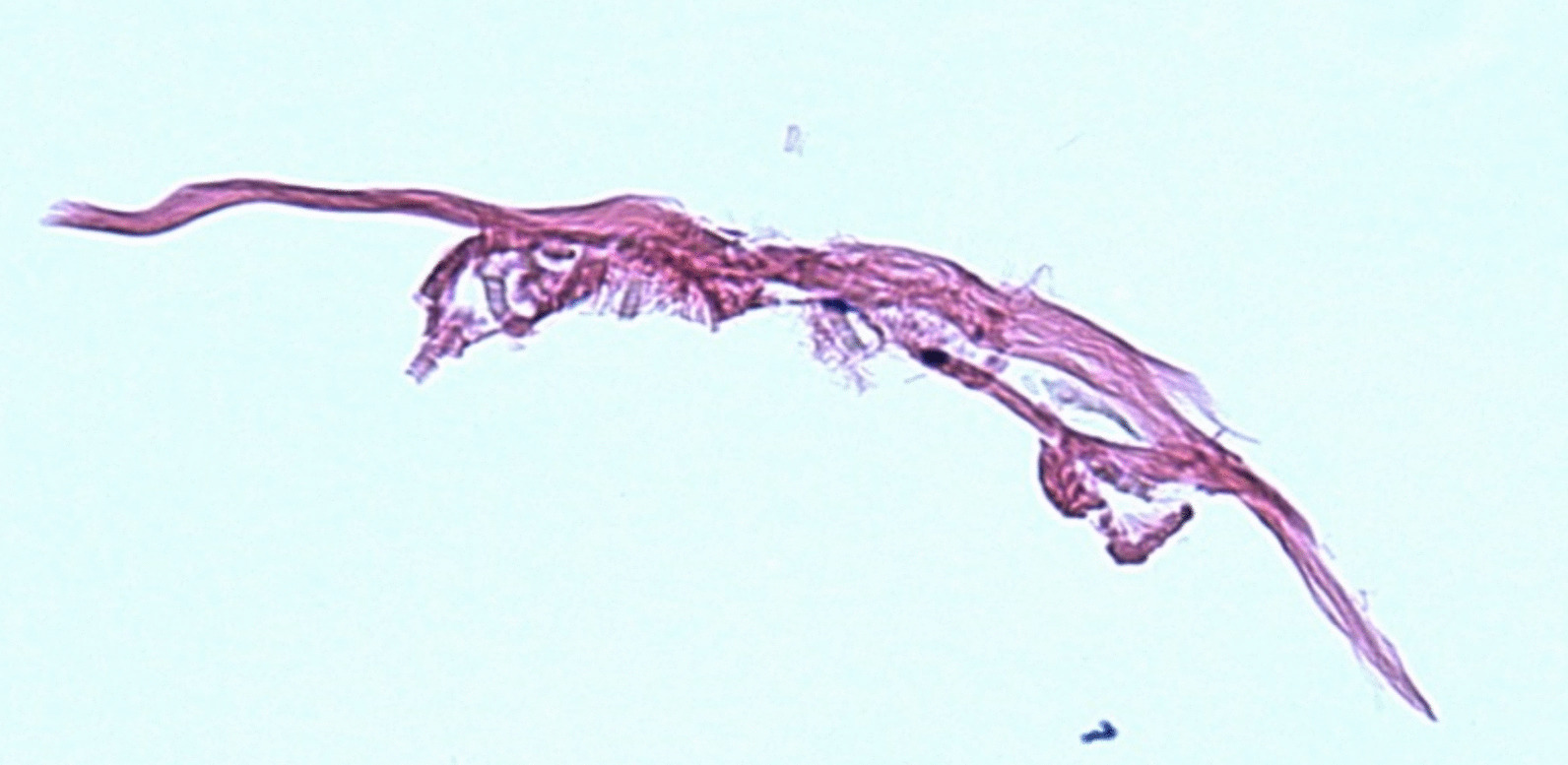
Punch biopsy from right anterior thigh showing histiocytes palisading around areas of mucin and degenerated collagen

Punch biopsy specimens from the anterior proximal thigh and the right anterior proximal upper arm both revealed dermal histiocytes palisading around areas of mucin and degenerated collagen, as seen in Fig. 4. The final dermatopathological diagnosis was granuloma annulare.

The dermatologist prescribed 0.1% triamcinolone acetanide topical cream twice daily and 500 mg metronidazole tablet twice daily for 10 days. After this, the patient’s lesions began to resolve gradually. After 3 months, they had completely gone away.

## Discussion

Relatively little is known about GA’s exact etiology. The clinical prognosis for GA varies according to clinical subtype. The localized variant generally resolves spontaneously within 2 years, whereas the generalized form is more chronic and less responsive to treatment [10].

Studies of successful and unsuccessful treatments for GA, although limited by size and their observational nature, give insight between the underlying pathology and therapeutic mechanism. The disease likely has an inflammatory component, and anti-inflammatory agents, such as corticosteroids, methotrexate, and sulphasalazine, alleviate the lesions [11–13]. It also possibly has an infectious etiology as dapsone, which is both an antibiotic and anti-inflammatory agent, has been shown as an effective treatment [14]. Granuloma annulare also occurs at sites of healing herpes zoster and verruca vulgaris lesions, lending the suggestion of a viral etiology [15]. Immunoglobulin-mediated vasculitis has also been suggested as one possible etiology in GA and may explain why pentoxifylline works to treat the disease [16, 17].

Our patient had the disseminated form of granuloma annulare characterized by widespread skin-colored or pinkish papules and plaque, usually arranged symmetrically in poorly defined rings 10 cm or more in diameter. Such lesions are often found around the skin folds of the trunk, armpits, and groin. Patients commonly complain of itching in the lesions.

Proposed causal mechanisms of subcutaneous GA include physical trauma, infections (streptococci, tuberculosis, Epstein–Barr virus, and herpes zoster), immunizations, insect bites, diabetes mellitus, and alterations in cell-mediated immune responses [18]. The presence of infection and chronic disease can worsen the prognosis of this uncommon condition [19]. Histopathology, this subtype of GA typically shows a deep dermal and/or hypodermal infiltrate of granulomas predominantly formed by palisaded histiocytes around a central region of degenerating collagen fibers and abundant mucin. Such features are best seen under alcian blue staining under the microscope [20].

Although in some cases the diagnosis of GA may be clinically straightforward, in other cases the initial clinical impressions can be either ambiguous or misleading making diagnosis difficult. The latter was the case in our patient. GA can resemble other granulomatous skin conditions [21]. The presence of mucin is an important histologic feature that favors the diagnosis of GA, since other granulomatous skin diseases, such as sarcoidosis and necrobiosis lipoidica, do not reveal mucin deposition [22]. Clinically, GA may be confused with many other skin diseases, including dermatofibroma, dermatomyositis, papulonecrotic tuberculid, psoriasis, tinea cruris, borreliosis, cutaneous borreliosis, Churg–Strauss Syndrome, Kaposi sarcoma, and tuberculoid leprosy [23–31].

The scientific strengths of our case report include laboratory testing to rule out diagnoses, as well as confirmatory histologic skin samples to confirm the diagnosis of GA. In the past, when active tuberculosis was more common, researchers believed that granuloma annulare was associated with or even caused by tuberculosis. In 1992, an inadequately treated case of active tuberculosis presented with clinical and histologic lesions compatible with granuloma annulare. After antituberculosis therapy was started, the skin lesions resolved [32]. Since tuberculin PPD is an inactivated purified protein fraction obtained from *Mycobacterium tuberculosis*, perhaps a similar relationship exists between PPD exposure and GA in our case, like that found between active tuberculosis and GA in prior studies. As with any case report, generalizing the validity of our study is limited, and we cannot establish a definite cause–effect relationship between PPD administration and GA with just a single case. However, we hope to at least raise the possibility of a causal effect that can be further explored in a future epidemiologic study.

## Conclusion

This case of subcutaneous GA was reported since it is a rare dermatologic pathological condition that can be confused with other skin rash disorders. This case is unique as it occurred after PPD administration. Our patient presented with erythematous papules and plaques without central clearing on her extremities and abdominal area 6 weeks after PPD administration. Punch biopsy specimens revealed dermal histiocytes palisading around mucin and degenerated collagen characteristic of GA. After treatment with triamcinolone acetanide and metronidazole, the patient’s lesions resolved. Although GA is a benign self-limited disease, definitive diagnosis is important to rule out other pathologies with similar clinical appearances, such as cancer or human immunodeficiency virus (HIV) infection. Diagnostic confirmation is best made through skin biopsy.

## Data Availability

Data contain potentially identifying or sensitive patient information. Contact information for a data access: Ernest Lee, MD, MPH ernest.lee1@va.gov

## Abbreviations

GA: Granuloma annulare
SARS: Severe acute respiratory syndrome
BCG: Bacillus Calmette-Guerin
PPD: Purified protein derivative
uL: Microliters
HIV: Human immunodeficiency virus
